# A Multimodal Pancreas Phantom for Computer-Assisted Surgery Training

**DOI:** 10.1109/OJEMB.2020.2999786

**Published:** 2020-06-03

**Authors:** Benjamin Eigl, Caroline Haslebacher, Philip C. Müller, Andreas Andreou, Beat Gloor, Matthias Peterhans

**Affiliations:** ARTORG Center for Biomedical Engineering ResearchUniversity of Bern 27210 Bern 3012 Switzerland; CAScination AG Bern 3008 Switzerland; CAScination Bern 3008 Switzerland; Physical Institute of the University of Bern 27210 Bern 3012 Switzerland; Department of Visceral and Transplant SurgeryUniversity Hospital Zurich 27243 Zurich 8091 Switzerland; Department of Visceral Surgery and MedicineUniversity Hospital of Bern 27210 Bern 3012 Switzerland; CAScination AG Bern 3008 Switzerland

**Keywords:** Artificial, computed-tomography, pancreas, phantom, ultrasound

## Abstract

Training of surgical residents and the establishment of innovative surgical techniques require training phantoms that realistically mimic human anatomy. Because animal models have their limitations due to ethical aspects, costs, and the required efforts to set up such training, artificial phantoms are a promising alternative. In the field of image-guided surgery, the challenge lies in developing phantoms that are accurate both anatomically and in terms of imaging properties, while taking the cost factor into account. With respect to the pancreas, animal models are less suitable because their anatomy differs significantly from human anatomy and tissue properties rapidly degrade in the case of ex vivo models. Nevertheless, progress with artificial phantoms has been sparse, although the need for innovative, minimally invasive therapies that require adequate training is steadily increasing. *Methods:* In the course of this project, an artificial pancreas phantom that is compatible with basic electrosurgical techniques was developed with realistic anatomic and haptic properties, computed tomography, and ultrasound imaging capabilities. This article contains step-by-step instructions for the fabrication of a low-cost pancreatic phantom. The molds are also available for download in a 3D file format. *Results:* The phantom was successfully validated with regard to its computed tomography and ultrasound properties. As a result, the phantom could be used in combination with a state-of-the-art computer-assisted navigation system. The resection capabilities were positively evaluated in a preclinical study evaluating endoscopic resections using the navigation system. Finally, the durability of the phantom material was tested in a study with multiple needle insertions. *Conclusion:* The developed phantom represents an open-access and low-cost durable alternative to conventional animal models in the continuous process of surgical training and development of new techniques.

## I. Introduction

Minimally-invasive surgery on the pancreas is of great interest because it reduces surgical morbidity typically associated with more invasive open surgery. These applications include laparoscopic resection and percutaneous ablation of pancreatic lesions. However, minimally-invasive interventions require highly experienced surgeons and interventional radiologists due to multiple critical structures surrounding the pancreas [1]–[3]. These, among others, include vascular structures such as the superior mesenteric artery, celiac artery (CA), portal vein (PV), and superior mesenteric vein, as well as the duodenum, common bile duct (CBD), and pancreatic duct. When applying minimally-invasive techniques, an excellent anatomical and spatial understanding of the anatomy is key to a successful treatment outcome. Computer assistance may increase the confidence and spatial understanding of the user, yet it requires dedicated models to master the application [4], [5].

Animal models bear significance for the training of surgical residents. However, there is a need for alternatives to learn basic laparoscopic or percutaneous techniques due to ethical queries and costs, especially for the in-vivo scenario [6]. Furthermore, ex-vivo models degrade rapidly due to autolysis. To establish innovative techniques, realistic phantoms are required that mimic the human anatomy. Although there have been significant advancements in modeling the human liver, achievements regarding artificial pancreas phantoms remain sparse [7], [8].

This work presents the development of a patient-realistic pancreas phantom that holds computed-tomography (CT) and ultrasound (US) properties and allows the application of basic electrosurgical techniques. These properties make it suitable for use with state-of-the-art computer-assisted navigation solutions. The pancreas is comprised of the pancreatic parenchyma with an incorporated pancreatic duct and intraparenchymal lesions. Furthermore, it is surrounded by vascular structures, the extrahepatic biliary structures, and the duodenum. The phantom was successfully evaluated during a proof-of-concept study on its ability to serve as a basis for computer-assisted resection and needle guidance.

## II. Materials and Methods

The underlying principle of the phantom was taken from Pacioni *et al.* [7], who describe the fabrication method of a polymer-based patient-specific liver phantom. Differentiation of the intraparenchymal structures (vessels and lesions) was possible due to the variation in echogenicity, yet the speed of sound in the various materials differed significantly from human tissue [7]. In contrast to polymers used in [7], the developed protocol described in [8] makes use of low-cost materials, such as agarose, and candle gel to mimic liver tissue.

Functional vasculature inside an artificial organ requires interconnected channels that replicate the vascular tree. This was achieved in [9], in which liquid wax was injected into the vessels of an ex-vivo human liver that was subsequently dissolved to keep the cast of only the vessels. The cast was placed into the liver mold, which was filled with the polymer and again melted after the curing process. This two-step casting process generated hollow structures to simulate any kind of liquid flow [9]. Respiratory motion was simulated in [10] using a linear servo actuator to add dynamic properties for liver biopsy training. Although the work does not focus on the development of the liver and its structures, it describes the process and models the motion control with the connected organ compartment [9]. Finally, a 3D-printed hollow pancreas was developed in [11] for single-photon emission computed tomography to evaluate various reconstruction settings.

Commercially available phantoms by Kyoto Kagaku (Kyoto Kagaku Co., Ltd., Japan) and CIRS (CIRS, Inc., US) represent the most advanced phantoms with respect to the number of intracorporal structures and imaging properties. However, the phantoms are designed for specific use cases such as percutaneous biopsy training or ultrasound training, which makes them less flexible for use in other settings. Based on the state-of-the-art methods from [7], [8], this study's pancreas phantom was developed and further optimized with respect to the US and CT properties and to fulfill the requirements regarding electrosurgical resections. The improvements were monitored with the intraoperative BK 5000 ultrasound (BK Medical, Denmark) and the Canon Aquilion CXL (Canon Medical, Japan). Furthermore, the surgical navigation system CAS-One (CAScination, Bern, Switzerland) was used to evaluate the pancreas regarding its capabilities in resection and needle navigation scenarios.

### A. Materials

The following materials were used to model the polymer-based phantom:

• Silicone (flexible, stable over time), Ecoflex 00-10 and Dragon Skin 10 Medium, Smooth-On, Inc., Macungie, Pennsylvania, US.
• Vaseline oil (increases echogenicity, reduces viscosity and ultrasound attenuation), Unicobres GmbH & Co. KG, Buchenau, Germany.
• Graphite powder (increases echogenicity; scattering), Kasp security, UK.
• Silicone Thinner (reduces viscosity), Smooth-On, Inc., Macungie, Pennsylvania, US.
• Slacker (responsible for self-healing properties), Smooth-On, Inc., Macungie, Pennsylvania, US.
• Barium sulfate (BaSO4, responsible for CT properties), PyroPowders, Erfurt, Germany.

Note that although the materials from Table I allow for the modeling of CT and US properties, silicone obtains a high melting point (above 200 °C), which is out of range for ordinary electrosurgical devices operating below 100 °C [12]. To produce a phantom suitable for resections, a pancreas out of agar-agar with the ingredients from Table II was evaluated.

**TABLE I:** Formulation For Preparing a Reference Mass of 100 g for a Desired Polymer-Based Structure

	Ecoflex A + B [g]	Dragon Skin A+ B [g]	Silicone Thinner [g]	Slacker [g]	Oil [g]	Graphite [g]	BaSO4 [g]
Pancreas	67.25	-	10	7	15	0.25	0.5
Tumor	55	-	10	7	22	5	1
Lesion	100	-	-	-	-	-	-
Surrounding Structures	-	94.5	5	-	-	-	0.5
Background	57	-	10	7	25	1	-

**TABLE II:** Formulation For Preparing a Reference Mass of 100 g for a Desired Agar-Based Structure

	Water [g]	Agar-Agar [g]	Corn starch [g]	Barium sulphate [g]
Pancreas	93	4	1	2
Lesion	94	5	0	0

Agar-based phantoms provide a low-cost alternative for ultrasound training [13]. The addition of barium sulfate enables the discrimination of the tumors from the parenchyma in the CT image data. The melting point between 80–85 °C makes it a potential candidate for electrosurgery [14].

### B. Modeling

In the course of the project, an artificial pancreatic phantom was developed that includes basic structures such as the pancreas, intrapancreatic lesions, gallbladder, aorta, CA, PV, CBD, and duodenum. Based on the pancreas 3D models obtained from Turbo Squid (New Orleans, Louisiana, US) [15], an inverse mold was derived, optimized, and 3D printed. The molds were printed with a 3D filament printer (Ultimaker 3, Ultimaker, Netherlands) with polylactic acid (PLA) filament.

The pancreas mold comprises two parts (anterior and posterior) with placeholders for multiple lesions (10 mm) at different heights, as well as a placeholder for the pancreatic duct. The remaining structures were casted from a six-part mold including the duodenum, aorta, gallbladder, and portal vein. Depending on the application, the pancreatic duct obtained a varying diameter and was stabilized with an artificial soft-tissue duct (LifeLike BioTissue, Inc., London, ON, Canada).

### C. Step-by-Step Production

**Fig. 1.**
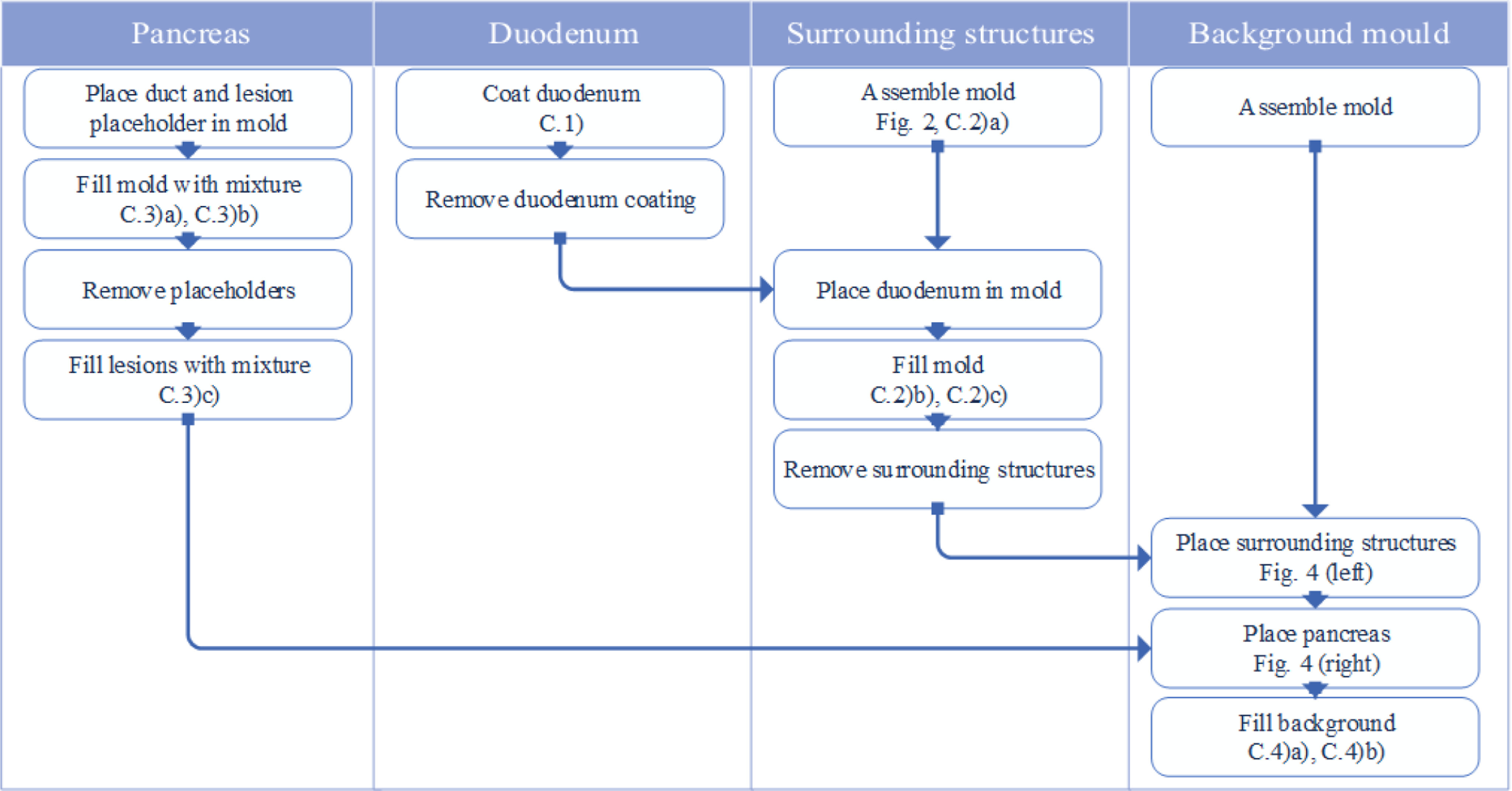
Flowchart of pancreas production process outlining the important steps.

Fig. 1 illustrates the production process. In addition to the printed 3D forms, the following tools are required:

– Gram scale
– Pan
– Whisk
– Syringe
– Tape and/or screw clamps

#### 1) Instructions For Hollow Duodenum

1) 3D print the section of the hollow duodenum.
2) Heat approximately 200 g of ComposiMold in a microwave oven in 45 second intervals with 300 watts.
3) Coat the 3D-printed part of the duodenum.
4) Wait 10–20 minutes until it has cooled down.
5) Cut open the ComposiMold to remove the 3D-printed part of the duodenum.
6) Heat some ComposiMold to fix the large holes that were created during cutting.
7) After cooling, place the duodenum section into mold part 5.

#### 2) Surrounding Structures

**Fig. 2.**
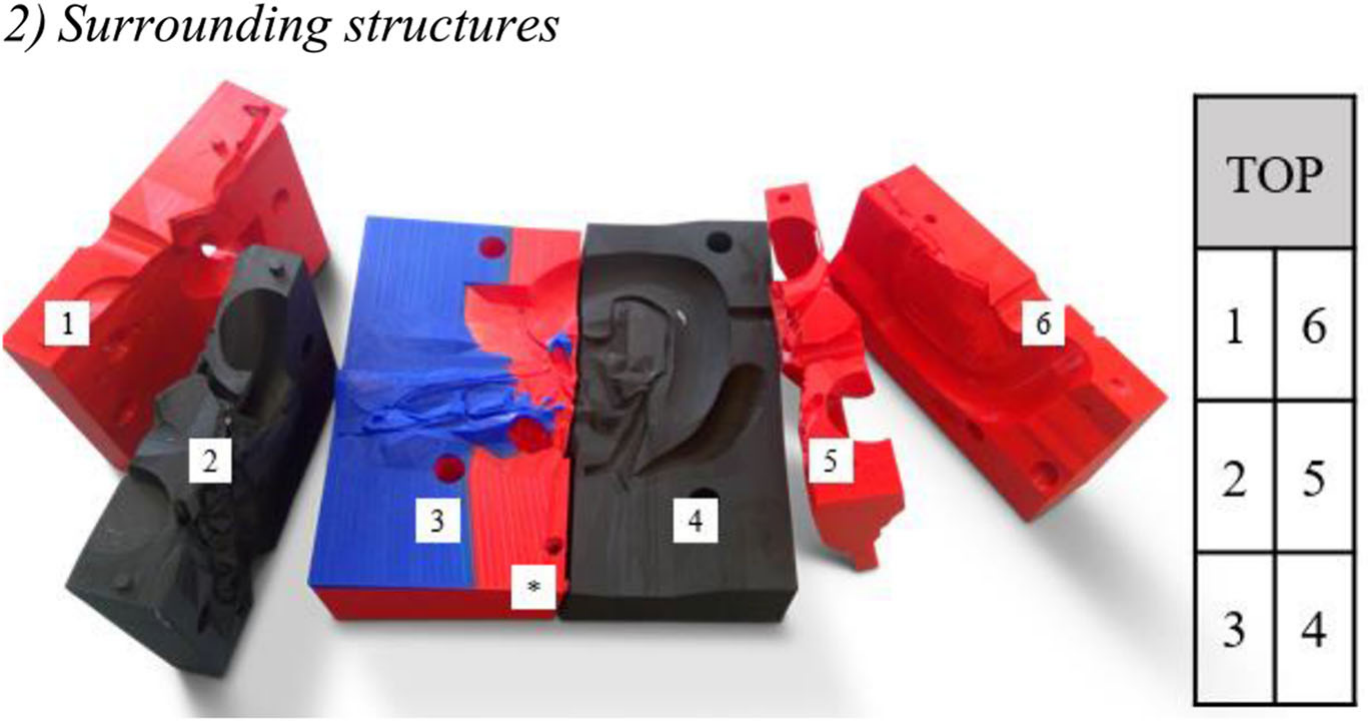
Numbered parts of the six-part mold for the surrounding structures (left); assembly order (right).

*INFORMATION:* Drill a hole into the lowest parts of the six-part mold (* in Fig. 2) to facilitate filling the aorta. Otherwise, due to the high viscosity of the silicone mixture, it may harden before the aorta is filled.

##### a) Assembly

1) Spray all parts of the mold with Ease-Release™ 200 (Smooth-On, Inc.) or brush on Vaseline oil to facilitate demolding.
2) Assemble the mold.
3) Use tape and/or screw clamps to seal the mold and prevent the mixture from flowing out.

##### b) Recipe For Polymer-Based Vascular Structures

1) Weigh 425 g Dragon Skin part A into a beaker with a volume of at least 2 liters.
2) Add 45 g Silicone Thinner and mix (e.g., with a wooden stir).
3) Add 425 g of Dragon Skin part B.
4) Mix thoroughly for 2–3 minutes.
5) Add 4.5 g barium sulfate.
6) Place the mixture into the vacuum chamber and activate the pump until the silicone expands to twice the size. Wait another 90 seconds before turning off the pump.

*IMPORTANT:* Do NOT take a long time to complete the following steps. The pot life of the mixture is about 25 minutes.

##### c) Filling Instructions for Surrounding Structures

*INFORMATION:* The aorta can be filled first and, once cured, the rest is filled. The silicone mixture bonds completely to the aorta.

1) Pour the mixture into the large hole for the duodenum and fill it to the brim (this takes a while).
2) If you want to use the hollow section of the duodenum, use the hole between parts 1 and 6 to fill the six-part mold.
3) Check that the mixture has filled the gallbladder and the portal vein. If not, pour the mixture into the corresponding holes.

*INFORMATION:* The mixture needs time (approx. 5 min) to flow through the mold to the bottom.

4) Make sure the aorta is filled to the top. If not, use the hole in part 3 to pour in more of the mixture.
5) Let it cure for 4 hours.
6) Remove the tape with a knife.
7) Use a flat-bladed screwdriver to carefully introduce air into the mold.
8) Remove the parts 1 and 6.
9) Cut the excess material with a knife. It is easiest at this stage. Be careful not to cut off small vessels if possible.

*INFORMATION:* As the mold is not 100% optimized, some vessels must be cut off to remove the structures. You can then affix them together with the same silicone mixture.

10) Loosen the bottom parts 3 and 4.
11) To remove the middle parts 2 and 5, carefully pull out the duodenum.
12) When the mold is successfully removed, cut away any excess material so the surroundings look clean.

#### 3) Pancreas

The following describes the production of an agar-agar-based pancreas with intraparenchymal lesions. If a polymer pancreas is desired, follow the recipe with the pancreas and lesion mixture from Table I.

##### a) Recipe for Agar-Based Pancreas

1) Mix 3.7 g of corn starch with approximately 10 ml cold water (otherwise, it will not dissolve).
2) Bring 372 g of water in a pan to boil.
3) Add 21.5 g of agar-agar.
4) Mix with a whisk.

*INFORMATION:* To thicken the mixture, increase the amount of agar-agar.

5) Add the corn starch mixture. Mix.
6) Add 7.5 g of barium sulfate. Mix.
7) Remove the pan from the stove and wait until the mixture has sufficiently cooled so the mold does not deform.

*INFORMATION:* The temperature should not be higher than 80 °C for a mold printed with PLA filament.

*INFORMATION:* If the temperature is too low, the mixture begins to harden and cannot be poured into the mold.

##### b) Filling Instructions for Pancreas

1) Insert the tumor placeholders.
2) Insert the pancreatic duct.
3) Seal the mold with tape (or drill holes for screws).
4) Pour the mixture into the dedicated filling hole.
5) Wait until the mixture has completely cooled to room temperature. If possible, wait overnight.

*INFORMATION*: Place the mold into a refrigerator or freezer to speed the cooling process.

6) Open the mold and cut off excess material.
7) Carefully remove the duct and tumor placeholders.

##### c) Recipe for the Intrapancreatic Lesions

1) Bring 96 g of water in a pan to boil.
2) Add 4 g of agar-agar and mix.
3) Remove the pan from the stove.
4) Fill the tumors with this mixture using the syringe.

*IMPORTANT*: Make sure to fill the spherical part where the tumor is located, and not the pillar parts.

5) Let it cool for one hour.
6) Mix 100 g of the pancreas mixture to fill the pillar parts.

*INFORMATION:* The pancreas will shrink over time due to dehydration. You can slow this process by vacuuming the finished pancreas with a food-vacuuming machine.

*INFORMATION:* To obtain a wider and more stable pancreatic duct, use a larger ductus placeholder in combination with LifeLike tissue. Immediately before use, LifeLike tissue is guided through the ductal placeholder with the help of a metal wire.

*INFORMATION:* LifeLike tissue may burn if inserted before the hot mix is poured in.

*INFORMATION:* A coating made of 100 g Dragon Skin and 1 g of THI-VEX can be brushed on the pancreas for stability.

#### 4) Finish

For stability, a background support can be 3D printed and can also serve as a mold for the background material. After choosing the height of the phantom, the mixture is produced according to the recipe from Table I.

##### a) Recipe for the Background Mixture

*INFORMATION:* To fill the phantom to the duodenal papilla, 900 g of the background mixture is needed. See Table I for material amounts.

1) Using a whisk, mix graphite and Vaseline oil in a beaker.
2) Pour silicone Ecoflex 00–10 part A into a beaker that is twice the volume of the total mixture.
3) Add Silicone Thinner.
4) Add silicone Ecoflex 00–10 part B.
5) Stir for 2–3 minutes.
6) Add the graphite-oil mixture. Mix.
7) Place the mixture into the vacuum chamber and turn on the pump until the silicone expands to twice the size. Wait another 90 seconds before turning off the pump.

##### b) Filling Instructions for the Background Mixture With Optional Mesenteric Tumor

*OPTIONAL:* To add the mesenteric tumor next to the celiac artery, prepare a tumor mixture according to the recipe from Table I and complete the steps from Section III-C.2)b) with the updated weights. Insert the tumor support into the connector next to the aorta and place the tumor on top.

1) Spray the box with Ease Release.
2) Place the structures into the box.

*IMPORTANT:* If the phantom is filled higher than up to the pancreatic papilla, the pancreatic duct must be occluded to prevent the background mixture from flowing in. Close the hole in the box next to the duodenum with tape or clay.

3) Pour the mixture slowly and let it cure for 4 hours.
4) The phantom is stable enough to be removed from the background support if necessary.

*Note:* If the mesenteric tumor was added, make sure to fill the placeholder hole with background mixture.

### D. Evaluation Methods

Throughout development, the pancreatic phantom has been used in various environments to evaluate imaging, resection properties, and needle insertion.

#### 1) Resection Properties

To evaluate the resection properties, the phantom was used in the course of a study on computer-assisted endoscopic resection of intraparenchymal tumors starting from the pancreatic duct [16]. The experimental technique of transduodenal-transpapillary endopancreatic surgery makes use of a hybrid NOTES approach in which a rigid endoscope is inserted into the pancreatic duct via a duodenal incision to perform a resection. The technique has been explored in human cadavers, as well as ex-vivo and in-vivo animal tests [17], and has been combined with computer assistance to enhance the localization of lesions not visible in the endoscopic image. This requires the display of anatomical landmarks in CT and US imaging, as well as resection capabilities. The study included four cases. Prior to each case, CT images of the phantom were acquired, and the structures segmented using a commercially available software (Myrian, Intrasense, Montpellier, France). To obtain computer-assisted resection guidance, the user performed a matching of landmarks acquired using a tracked ultrasound probe with the respective landmarks in the image data [16].

#### 2) Needle Insertion

To evaluate the suitability for interventional training, a study was conducted in which computer-assisted needle placements were performed. This study made use of a simplistic, polymer-based version of the phantom with an aorta and a tumor encompassing the celiac artery. The background mixture was used to hide the internal structures from the user. The study compared needle guidance approaches using a state-of-the-art navigation system for interventional radiology (CAS-One IR, CAScination AG, Bern Switzerland). The participants were requested to place three needles around the tumor under computer-assisted guidance, with and without the aid of a mechanical arm [18].

## III. Results

The resulting pancreas phantom is illustrated in Fig. 4(a) and required a total working time of 5 hours with material costs of 100 EUR, excluding the costs for the molds. The background box had a significant value in generating several phantoms with constant relative positions of the main structures. The silicone-based structures retained their original shape over time while the agar-based material was subject to a high dehydration rate.

### A. CT Imaging

**Fig. 3.**
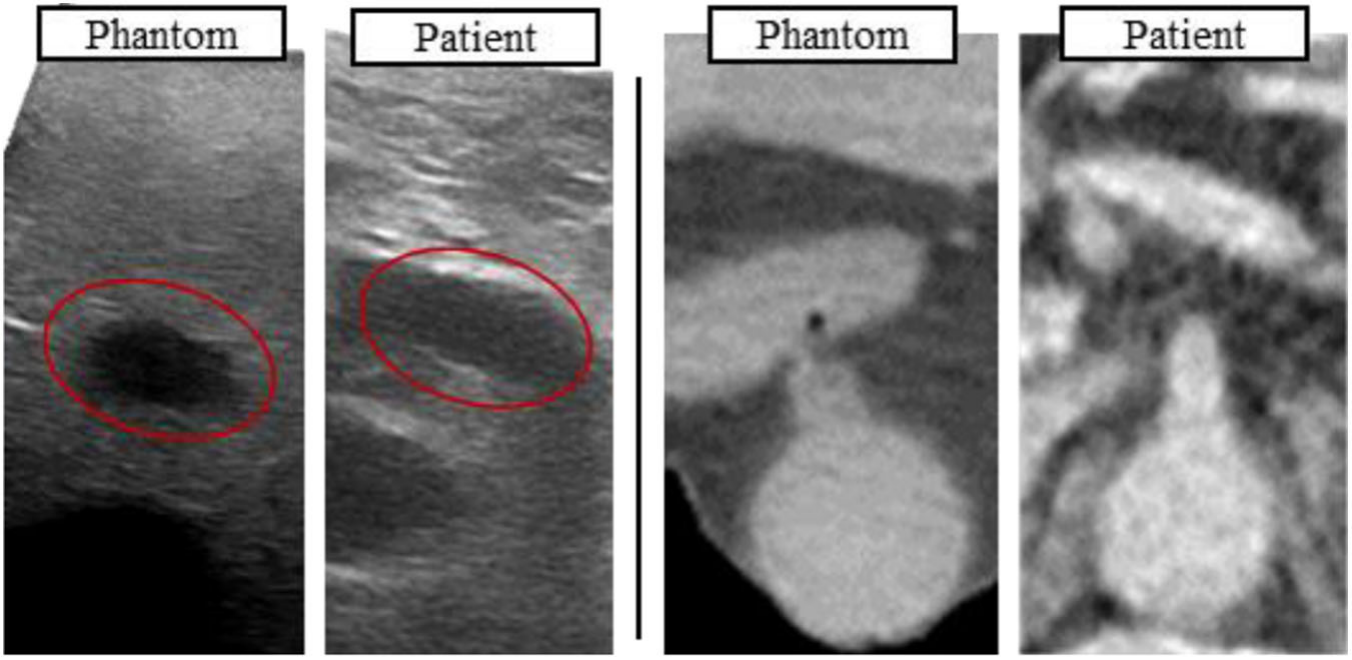
Comparison of imaging properties: (left) US image of hepatic artery (red outline) with patient US image acquired during open pancreas surgery; (right) cropped CT image of Aorta at SMA branch (patient CT scanned in PV phase).

**Fig. 4.**
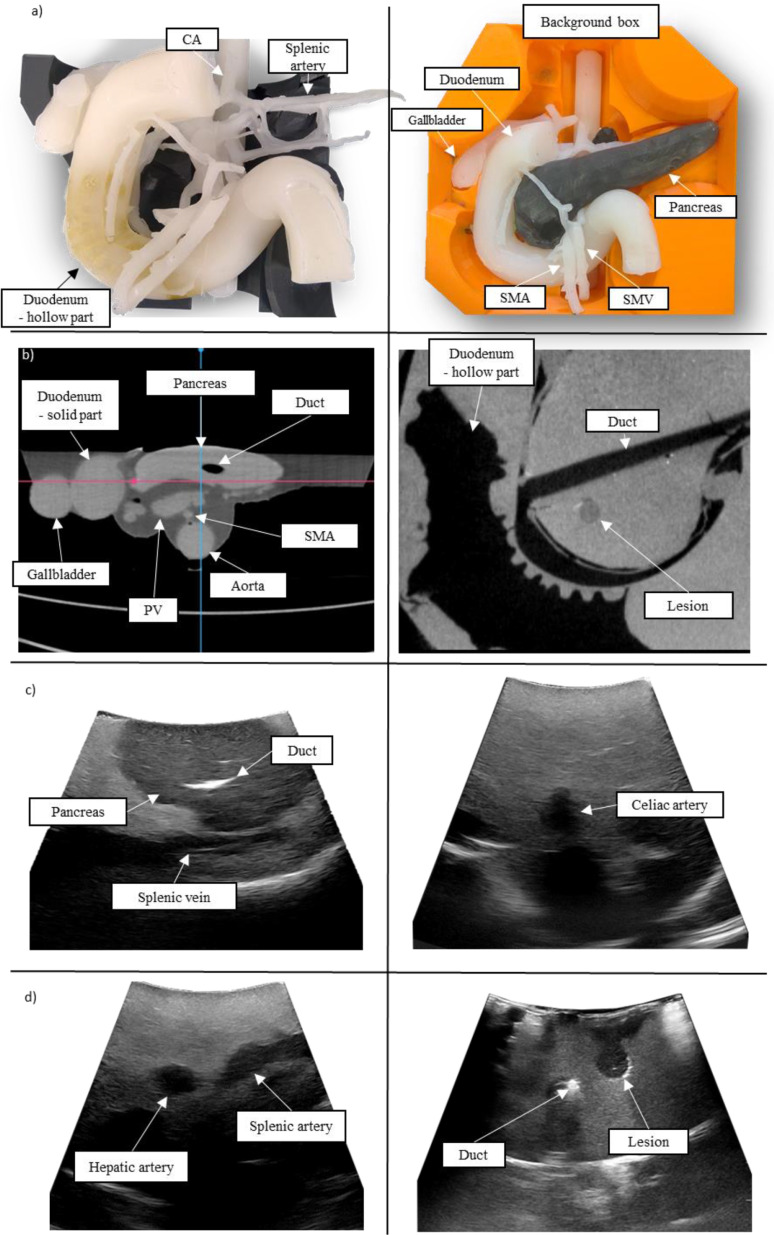
Results depicting: a) physical models of the pancreas phantom, b) CT images of plain polymer phantom axial (left), coronal (right), and c-d) US images of internal structures.

The use of barium sulfate allowed for the differentiation of the structures from their local environment, although the Hounsfield units do not represent realistic values (Fig. 3 and Fig. 4(b)).

### B. US Imaging Results

The differentiation of the structures from their local environment was possible, although the attenuation of the materials was excessively high, which limited the penetration depth of the ultrasound at a frequency of 5 MHz (Fig. 3 and Fig. 4(c), (d)). The speed of sound in the silicone material was lower than in human tissue, which affected the US image scaling.

#### 1) Resection Properties Results

**Fig. 5.**
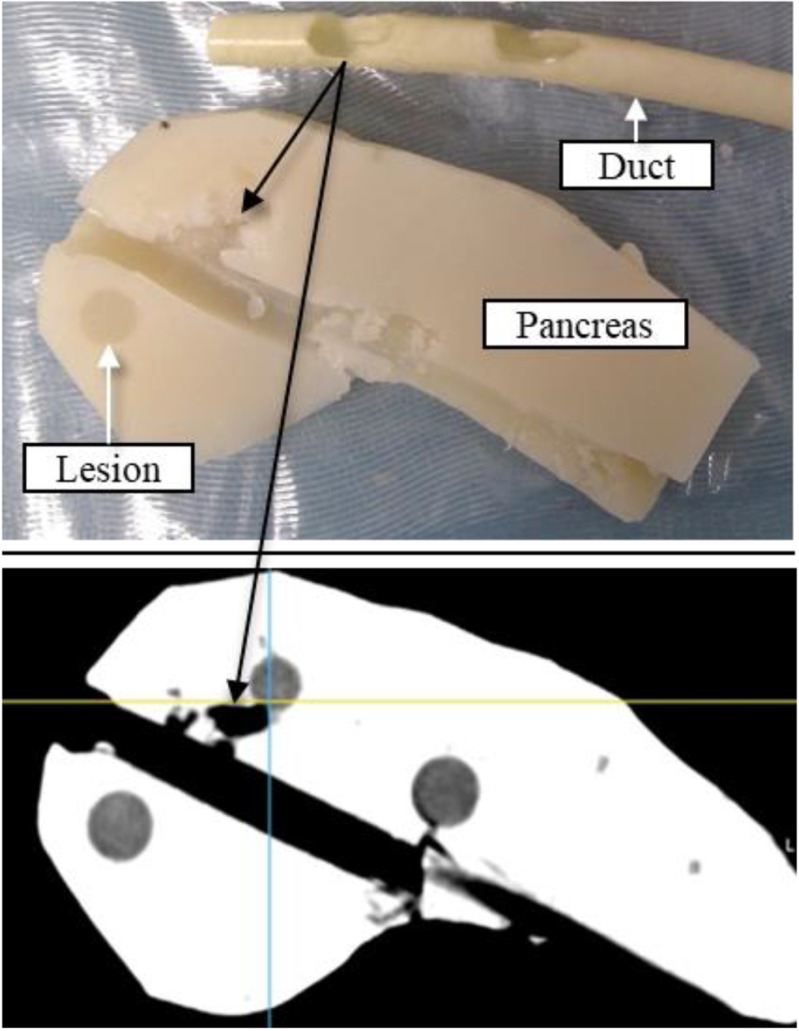
Resected path from the duct (start of black arrows) toward the lesion in the agar-based phantom. Axial cut (top); CT slice (bottom).

The agar-agar-based pancreatic phantom with the LifeLike duct could be resected endoscopically. During the study, 4 pancreatic phantoms were produced and a total of 7 resection attempts were performed, 6 of which led to successful resection from the duct toward the lesion (Fig. 5). The physician was able to identify the structures in the ultrasound and CT images that were needed for computer-assisted resection. Detailed explanations of the experiment can be found in the publication by Müller *et al.* [16].

#### 2) Needle Insertion Results

**Fig. 6.**
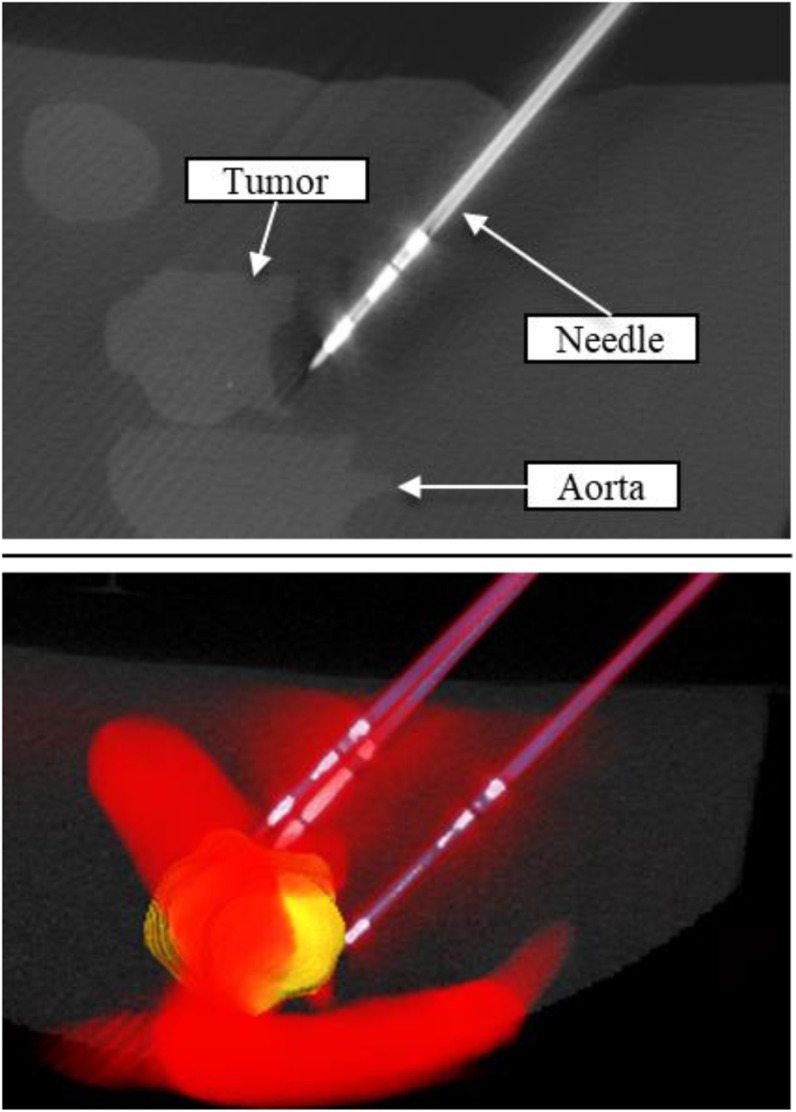
Placed ablation needles around tumour. CT slice (top), volume rendering (bottom).

Two simplistic box phantoms were created to evaluate two needle guidance approaches. The study group consisted of 7 participants with a total of 21 needle insertions per phantom. The durability of the phantom was successfully evaluated, and it was found that the material was only marginally affected by repeated punctures. The CT imaging provided the ability to differentiate between the tumor and the needles (Fig. 6). The main finding of the study was the increased spatial accuracy of needle insertion using the mechanical arm compared to the manual approach. Further details of the experiment can be found in the publication [18] (currently in review).

## IV. Discussion

This is the first study using a pancreatic phantom to combine essential surrounding structures with properties for ultrasound and CT imaging and to evaluate basic electrosurgical procedures. The anatomical details can range from patient-specific to more general models. The more detailed the anatomical structures, the more work is required in terms of design and fabrication.

The phantom was suitable to test the concept of a new resection technique of pancreatic lesions in combination with computer-assisted resection guidance. The electrosurgical properties enabled the resection from the pancreatic duct to the intraparenchymal lesions. The modular principle of the pancreas phantom allows easy replacement of the pancreas itself. The background box allows the production of multiple phantoms with constant relative position of the main structures. The polymer-based environment further improves the durability of the phantom, and the agar-based materials complement the resection properties. The combination of both materials did not deteriorate the CT and US imaging properties of the phantom as long as there is no air trapped between the structures. Because the speed of sound in silicone materials is comparatively lower than in human tissue (1080 m/s versus 1540 m/s), the US scale is affected [7]. Due to the high amount of water in the agar-based material, the speed of sound converges towards the desired speed, however the material is subject to dehydration over time [19]. In contrast, the silicone does not suffer from a high rate of dehydration and thus retains its original shape over a longer period of time [7]. To overcome this loss of accuracy, up-to-date CT images with subsequent segmentation are recommended to represent the actual state of the phantom.

The phantom was suitable for use in a computer-assisted needle navigation study and could be used in the future for training in interventional radiology or interventional pancreatic surgery. Further applications for the phantom could be seen in the training of surgical residents to improve their skills in handling the pancreas, such as during a pancreaticojejunostomy.

More optimization is needed to increase the ultrasound penetration depth, the speed of sound, and to accurately mimic the Hounsfield Units of human tissue. The resection was compromised by the fragility of the materials used and would need to be further improved. Furthermore, the resistance of the polymer during needle insertion was excessively high due to the lack of optimization of tissue-specific density replication.

Poly (vinyl alcohol) cryogel (PVA-C) as a tissue mimicking material represents a potential alternative to the agar-agar mixture. While PVA-C has been investigated for use in ultrasonic and magnetic resonance imaging, barium sulfate would complement the CT properties. In addition, resections could be feasible due to the large amount of water [20].

## V. Conclusion

In summary, we have presented the development of a multi-purpose pancreatic phantom including surrounding vessels and tissue using a detailed manufacturing process. The agar-based materials enable electrosurgical material removal, and the built-in pancreatic duct is stable enough to be explored with an endoscope. This phantom can be seen as a starting point for the creation of patient-specific pancreatic phantoms to train residents, optimize surgical or interventional techniques, and prove the concept of innovative pancreatic cancer treatments.

## Supplementary Material

10.21227/ph5s-hn75 Mold Materials for an Artificial Pancreas Phantom This data set contains all relevant 3D mold files for modeling a pancreatic phantom described in the publication: Benjamin Eigl et al.: A Multimodal Pancreas Phantom for Computer-Assisted Surgery Training. https://ieeexplore.ieee.org/document/9107339 https://ieee-dataport.org/open-access/mold-materials-artificial-pancreas-phantom
